# Burden of Mesothelioma in China, 1990–2023: Trends, Decomposition, and Projections Until 2045

**DOI:** 10.3390/cancers18152521

**Published:** 2026-08-06

**Authors:** Kang Hu, Qichen Ye, Rongrong Zhao, Chao Ma, Xiao Zhang, Tianhao Xie, Chenye Shao, Cheng Ding, Jun Zhao, Hao Ding

**Affiliations:** 1 Institute of Thoracic Surgery, Institute of Minimally Invasive Thoracic Cancer Therapy and Translational Research, The First Affiliated Hospital of Soochow University, Soochow University, Suzhou 215123, China; 2 Department of Thoracic Surgery, The First Affiliated Hospital of Soochow University, Soochow University, Suzhou 215123, China; 3 Department of Pharmaceutics, College of Pharmaceutical Sciences, Soochow University, Suzhou 215123, China; 4 Department of Radiology, Clinical Medical College, Peking University People’s Hospital, Beijing 100044, China

**Keywords:** mesothelioma, Global Burden of Disease Study 2023, disability-adjusted life years, age-standardized rates, joinpoint regression, decomposition analysis

## Abstract

**Background**: Mesothelioma is a rare but highly aggressive malignancy strongly associated with asbestos exposure. Owing to its long latency and poor prognosis, its burden requires systematic evaluation. **Methods**: Data on prevalence, incidence, deaths, disability-adjusted life years (DALYs), and age-standardized rates were extracted from the Global Burden of Disease Study 2023. The estimated annual percentage change, Joinpoint regression, Das Gupta decomposition, and Nordpred forecasting were used to assess temporal trends, identify turning points, quantify demographic and epidemiological contributions, and project future burden through 2045. **Results**: From 1990 to 2023, the absolute burden of mesothelioma in China increased substantially. Prevalent cases rose by 189%, incident cases by 150%, DALYs by 98%, and deaths by 142%. Males consistently showed a higher burden than females, and the burden was concentrated mainly among middle-aged and older adults. The age-standardized prevalence rate and age-standardized incidence rate increased, whereas the age-standardized DALY rate and age-standardized mortality rate remained stable or declined slightly. Decomposition analysis indicated that population growth and aging were the principal drivers of increased DALYs and deaths, while epidemiological change contributed negatively. Projections suggested that deaths may continue to increase through 2045, despite declining age-standardized fatal burden. **Conclusions**: This is the first update of the burden of mesothelioma in China over the past thirty-four years. The absolute burden of mesothelioma in China, as estimated by the GBD study, increased markedly, largely driven by demographic changes. Strengthening asbestos exposure surveillance, diagnostic standardization, and cancer registration systems would enable burden estimates to be derived from directly observed and certified data rather than relying primarily on model-based assumptions, while potentially identifying previously unrecognized sources of asbestos exposure.

## Simple Summary

From 1990 to 2023, China’s mesothelioma burden rose sharply, with cases up 189% and deaths up 142%, mainly affecting older males. While age-standardized death and disability rates stabilized or fell, population growth and aging drove the overall increase, and deaths are projected to keep rising through 2045. The study calls for better asbestos surveillance, diagnostics, and cancer care to reduce future impact.

## 1. Introduction

In recent decades, mesothelioma, a rare but highly aggressive malignancy, has gradually become an important issue in occupational health and public health [1]. Mesothelioma mainly arises from mesothelial cells of the pleura, peritoneum, pericardium, and tunica vaginalis, with malignant pleural mesothelioma being the most common subtype [2]. Although its overall incidence is relatively low, mesothelioma is characterized by high malignancy, a long latency period, and nonspecific early symptoms [3]. Patients are often diagnosed only after developing symptoms such as chest pain, dyspnea, pleural effusion, fatigue, or weight loss, and many cases are already locally advanced or advanced at the time of diagnosis [4]. Consequently, mesothelioma is generally associated with poor prognosis and limited treatment options, imposing a substantial burden on patients’ quality of life, family caregiving, and healthcare systems [5].

Asbestos exposure is the most well-established and important risk factor for mesothelioma [6]. Because mesothelioma may develop several decades after initial exposure, its current disease burden often reflects the long-term consequences of past industrialization, the use of construction materials, occupational protection practices, and environmental exposure control [7]. Even in countries and regions where asbestos use has been gradually restricted, the health effects of historical exposure may continue to emerge. In addition to occupational exposure, environmental asbestos contamination, secondary household exposure, residual asbestos-containing materials in old buildings, and inadequate exposure monitoring may further influence disease risk across different populations and regions [8].

As a country with a large population, China faces a mesothelioma burden of particular public health significance [9,10]. With accelerating population aging, the gradual manifestation of past occupational and environmental exposures, and continuous improvements in diagnostic capacity, the incidence and mortality burden of mesothelioma in China may continue to change over time [11]. However, because mesothelioma is relatively rare, clinical recognition and epidemiological surveillance remain insufficient, and systematic evaluation is still needed to clarify changes in disease burden across age groups, sexes, and time periods.

Based on data from the Global Burden of Disease Study 2023 (GBD 2023), this study systematically evaluated temporal trends in the prevalence, incidence, mortality, and disability-adjusted life years (DALYs) of mesothelioma in China from 1990 to 2023, and characterized its disease burden by sex, age, and time. This study aims to provide scientific evidence for identifying key populations for prevention and control, strengthening the management of asbestos-related exposures, improving early diagnostic capacity, and developing prevention and control strategies for mesothelioma.

## 2. Methods

### 2.1. Data Sources and Disease Definition

Data for this study were obtained from the GBD 2023 (Institute for Health Metrics and Evaluation, Seattle, WA, USA). GBD 2023 systematically estimated disease burden by country, region, age, sex, and year from 1990 to 2023, covering 204 countries and territories, 660 subnational locations, 375 diseases and injuries, and 88 risk factors [12]. GBD uses a standardized framework for data integration, disease modeling, bias correction, and uncertainty estimation to improve the comparability of estimates across time and locations. All estimates are reported with 95% uncertainty intervals (UIs), calculated from the 2.5th and 97.5th percentiles of 1000 posterior draws [13]. Mesothelioma is an aggressive malignant tumor originating from mesothelial cells, primarily involving the pleura, but it may also occur in the peritoneum, pericardium, and tunica vaginalis. In GBD 2023, mesothelioma is defined and classified according to the ICD-10 code C45 [10].

This study extracted and analyzed the number of prevalent cases, incident cases, deaths, DALYs, and corresponding age-standardized rates (ASRs) of mesothelioma in China from 1990 to 2023, including the age-standardized prevalence rate (ASPR), age-standardized incidence rate (ASIR), age-standardized mortality rate (ASMR), and age-standardized DALY rate (ASDR) [14,15,16]. DALYs consist of years lived with disability (YLDs) and years of life lost due to premature mortality (YLLs), namely DALY = YLD + YLL.

All rates (prevalence, incidence, mortality, and DALYs) were calculated using the total population of China as the denominator, stratified by age and sex. Rates are expressed per 100,000 population in the corresponding age-sex group. ASRs were calculated using the GBD standard population—a modified world standard population developed for the Global Burden of Disease Study—to ensure comparability across countries and over time. This standard population is derived from the global age structure and is applied consistently across all GBD analyses. The ASR for each indicator was calculated as the weighted average of age-specific rates, where the weights are the proportions of the standard population in each age group.

### 2.2. Data Quality and Modeling in the GBD Framework for China

The GBD 2023 estimates for China are generated using a standardized modeling framework that integrates multiple data sources. Primary data sources include the Chinese National Cancer Registry, the National Vital Registration System, published epidemiological studies, and hospital-based cancer registry data. For cancer incidence, GBD uses a mortality-to-incidence ratio (MIR) approach or directly uses cancer registry data when available. For mortality estimation, the GBD employs the Cause of Death Ensemble model (CODEm), which selects the optimal model among multiple competing approaches based on out-of-sample predictive validity. In settings with incomplete data, estimates are informed by covariates including health system access, income per capita, educational attainment, and other relevant predictors.

For mesothelioma specifically, several data quality considerations are relevant. Mesothelioma is a rare disease that may be underdiagnosed or misclassified, particularly in regions where pathological diagnostic services and cancer registration systems are limited. It may be misclassified as other pleural malignancies, lung cancer, or unspecified cancers of the respiratory tract. The GBD framework includes systematic adjustments for misclassification and redistribution of ‘garbage codes’ (non-specific causes of death that cannot be used as underlying causes). Estimates are accompanied by 95% uncertainty intervals (UIs), calculated from the 2.5th and 97.5th percentiles of 1000 posterior draws, which reflect both sampling variability and uncertainty from model assumptions and data limitations. Regional variations in data quality within China—with more comprehensive data typically available from urban and coastal regions compared to rural and inland areas—may affect the precision of national estimates. Readers should interpret the results with these data quality considerations in mind.

### 2.3. Trend Analysis

Temporal trends in China’s mesothelioma ASRs were analyzed using the estimated annual percentage change (EAPC) [17]. A log-linear regression model was applied: ln(ASR) = α + β × calendar year + ε. The EAPC formula is: EAPC = 100 × (eβ − 1). An EAPC above 0 signifies an upward trend, while below 0 indicates a downward trend.

### 2.4. Joinpoint Regression Analysis

Joinpoint regression analysis was used to assess long-term trends in the age-standardized disease burden rates of mesothelioma in China from 1990 to 2023, including ASPR, ASIR, ASMR, and ASDR [18]. This method identifies statistically significant turning points in time-series data, thereby describing the rate of change in disease burden across different periods. The analysis was performed using the Joinpoint Regression Program developed by the National Cancer Institute (version 5.2.0; Bethesda, MD, USA). A maximum of five joinpoints was allowed, and the optimal model was determined based on permutation tests and the Bayesian Information Criterion (BIC). Segment-specific trends were expressed as annual percent change (APC), while the overall trend across the entire study period was expressed as average annual percent change (AAPC).

### 2.5. Decomposition Analysis

Das Gupta decomposition analysis was used to quantify the relative contributions of population growth, population aging, and epidemiological change to changes in mesothelioma DALYs and deaths in China [19]. By constructing counterfactual scenarios, this method decomposes the total change from 1990 to 2023 into the independent effects of different driving factors. Population growth reflects the impact of changes in population size; population aging reflects the impact of changes in age structure; and epidemiological change represents the effect of changes in age-specific disease burden rates after controlling for population size and age structure. This component may be associated with historical asbestos exposure, occupational protection, diagnostic capacity, treatment accessibility, and survival improvement.

### 2.6. Forecasting Analysis

The Nordpred model, based on an age-period-cohort framework, was employed to forecast mesothelioma trends in China for the next 20 years [20]. This model is based on an age-period-cohort framework that integrates age effects, period effects, and birth cohort effects. The model assumes that historical trend patterns, including age-specific rates and cohort effects, will continue into the future. In the projection process, it is assumed that the major factors influencing the burden of mesothelioma—such as diagnostic capacity, treatment effectiveness, healthcare accessibility, and population health status—will evolve gradually, without abrupt structural changes. Although the model incorporates future changes in the age and sex structure of the population, it cannot fully anticipate future disease incidence risks, therapeutic breakthroughs, or adjustments in healthcare policies. Therefore, the projection results should be interpreted as scenario-based forecasts grounded in current trend assumptions, rather than as precise estimates of the actual future burden.

### 2.7. Software

The following software was used in this study: Joinpoint Regression Program (version 5.2.0; National Cancer Institute, Bethesda, MD, USA) for joinpoint regression analysis; R software (version 4.3.0; R Foundation for Statistical Computing, Vienna, Austria) for statistical computing, EAPC calculation, and Nordpred projection modeling; and the Nordpred package (version 1.0; Cancer Registry of Norway, Oslo, Norway) implemented in R for age-period-cohort forecasting.

## 3. Results

### 3.1. Burden of Mesothelioma

Over the past 34 years, the prevalence of mesothelioma in China increased from 1607.49 cases (95% UI: 1219.50 to 2116.23) in 1990 to 4640.31 cases (95% UI: 3545.91 to 6025.91) in 2023, representing a 189% increase. The percentage increase was 221% in males and 130% in females. The number of incident cases increased by 150% over the same period, with increases of 160% in males and 135% in females. DALYs due to mesothelioma increased from 37,848.71 (95% UI: 28,703.04 to 50,106.38) in 1990 to 74,980.69 (95% UI: 57,750.03 to 95,028.23) in 2023, representing a 98% increase. The increase was 108% in males and 84% in females. Deaths increased by 142% over the past 34 years, with increases of 141% in males and 142% in females.

The ASDR of mesothelioma changed from 3.81 per 100,000 population (95% UI: 2.89 to 5.04) in 1990 to 3.46 per 100,000 population (95% UI: 2.67 to 4.36) in 2023, with an EAPC of −0.21 (95% CI: −0.38 to −0.03). The EAPCs for ASPR, ASIR, and ASMR were 0.83 (95% CI: 0.65 to 1.01), 0.20 (95% CI: 0.02 to 0.37), and −0.03 (95% CI: −0.20 to 0.14), respectively (Table 1).

### 3.2. Age and Sex Distribution of Mesothelioma

**Figure 1:**
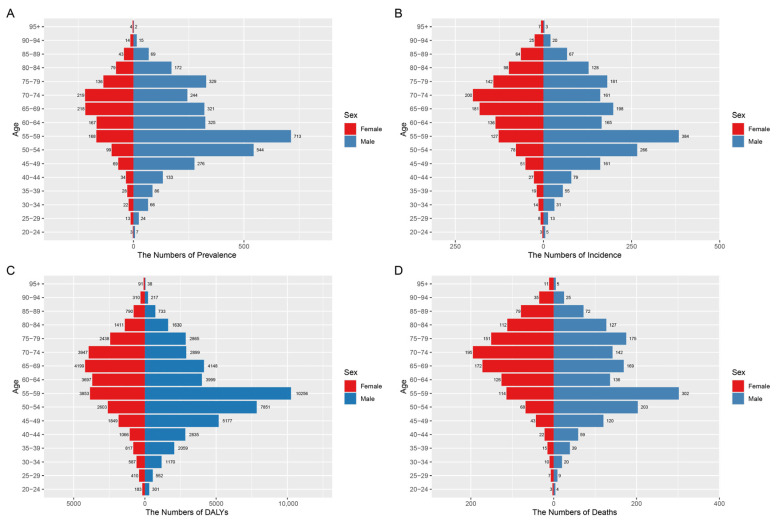
Age- and sex-stratified numbers of mesothelioma in China, 2023: (**A**) prevalence; (**B**) incidence; (**C**) DALYs; (**D**) deaths.

In 2023, among Chinese males, the highest numbers of mesothelioma prevalence, incidence, DALYs, and deaths were observed in the 55–59 age group, with 713, 384, 10,256, and 302 cases, respectively. Among Chinese females, the highest numbers of prevalence, incidence, and deaths were observed in the 70–74 age group, with 219, 200, and 195 cases, respectively, while the highest number of DALYs was observed in the 65–69 age group, at 4199 (Figure 1A–D).

**Figure 2:**
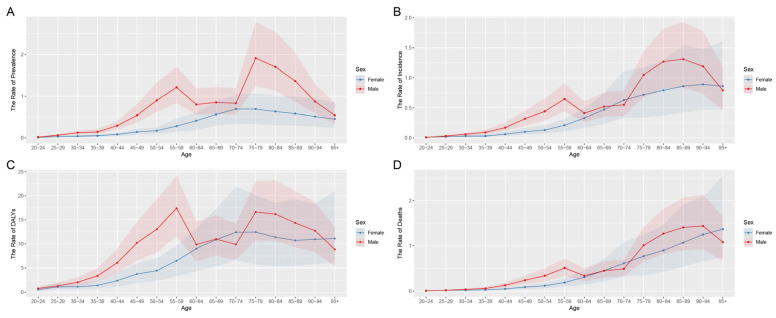
Age-specific rates of mesothelioma in China in 2023, per 100,000 population: (**A**) prevalence; (**B**) incidence; (**C**) DALYs; (**D**) mortality.

Figure 2A–D show that, among males, the prevalence rate was highest in the 75–79 age group, the incidence rate was highest in the 85–89 age group, the DALY rate was highest in the 55–59 age group, and the death rate was highest in the 90–94 age group. Among females, the death rate increased with age. Across most age groups, the rates were markedly higher in males than in females.

### 3.3. Trends over Time for Mesothelioma

From 1990 to 2023, the burden of mesothelioma in China showed a male-dominant sex pattern. The numbers of prevalent cases, incident cases, DALYs, and deaths generally increased over time. Male case numbers were consistently higher than female case numbers, and the sex gap gradually widened over the study period. The increase in case numbers was relatively gradual in the early period, followed by a faster rise, reaching relatively high levels in recent years.

**Figure 3:**
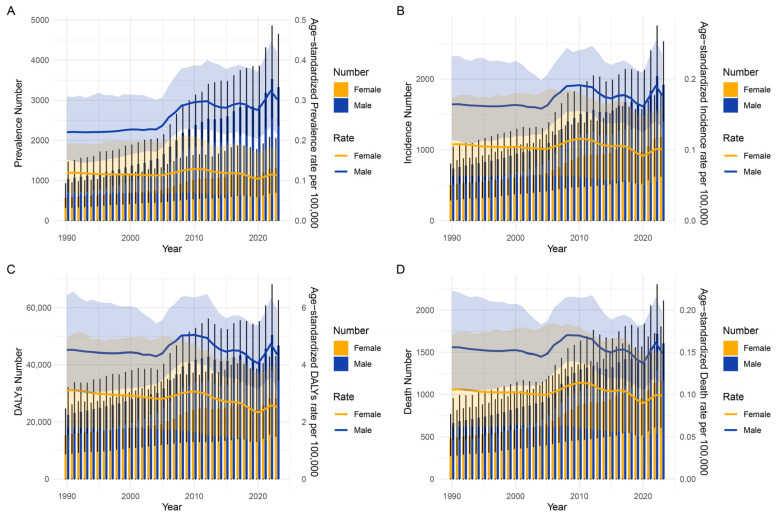
Trends in age-standardized rates and case numbers of mesothelioma in China from 1990 to 2023, by sex: (**A**) prevalence; (**B**) incidence; (**C**) DALYs; (**D**) deaths.

Trends in ASRs were not fully consistent with trends in case numbers. ASPR and ASIR showed overall fluctuating upward trends during the study period, with male rates consistently higher than female rates and showing more pronounced increases in the middle and later periods. In contrast, the ASDR and ASMR increased from the early to middle periods and then showed some fluctuation or decline, while remaining at relatively high levels in recent years. ASRs among females were generally lower than those among males, with smaller changes over time (Figure 3A–D).

### 3.4. Age-Specific Burden of Mesothelioma

**Figure 4:**
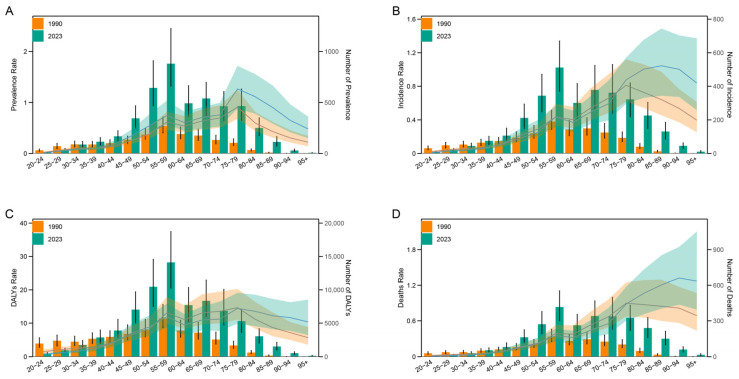
Comparison of age-specific numbers and rates of mesothelioma in China between 1990 and 2023: (**A**) prevalence; (**B**) incidence; (**C**) DALYs; (**D**) deaths.

From 1990 to 2023, both the number of cases and rates of mesothelioma increased overall across age groups in China, with differences mainly concentrated among middle-aged, older, and elderly populations. In 2023, the numbers of prevalent cases, incident cases, DALYs, and deaths were all markedly higher than those in 1990, with peak case numbers mostly occurring after the age of 50 years. Correspondingly, the rates of all indicators also shifted upward compared with 1990 and increased with age, with particularly prominent differences in older age groups. In contrast, younger age groups maintained a relatively low burden in both 1990 and 2023 (Figure 4A–D).

### 3.5. Joinpoint Regression Study on Mesothelioma Trends in China Between 1990 and 2023

**Figure 5:**
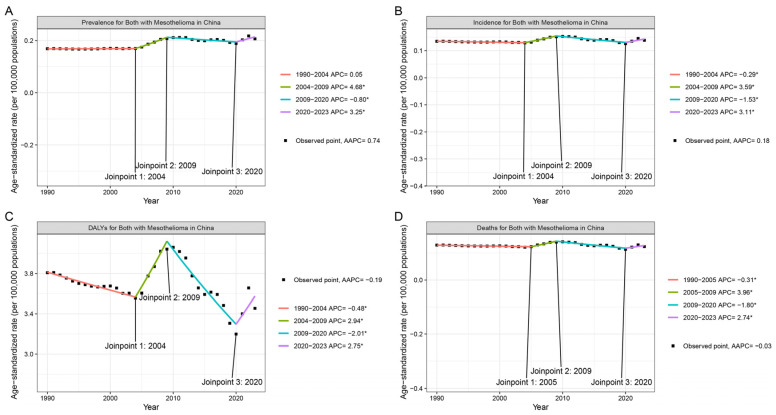
Analysis using joinpoint regression on age-standardized rates for prevalence, incidence, DALYs, and mortality from mesothelioma in China between 1990 and 2023. (**A**) ASPR. (**B**) ASIR. (**C**) ASDR. (**D**) ASMR. * The Annual Percent Change (APC) is significantly different from zero at the alpha = 0.05 level.

Joinpoint regression analysis showed that the ASPR and ASIR of mesothelioma in China increased significantly from 1990 to 2023, with AAPCs of 0.74 and 0.18, respectively (Figure 5A,B). In contrast, ASDR and ASMR showed decreasing trends, with AAPCs of −0.19 and −0.03, respectively (Figure 5C,D). Specifically, the fastest increases in ASPR, ASIR, and ASDR occurred during 2004–2009, with APCs of 4.68, 3.59, and 2.94, respectively. The fastest increase in ASMR occurred during 2005–2009, with an APC of 3.96.

### 3.6. Decomposition of Changes in Mesothelioma DALYs and Deaths

**Figure 6:**
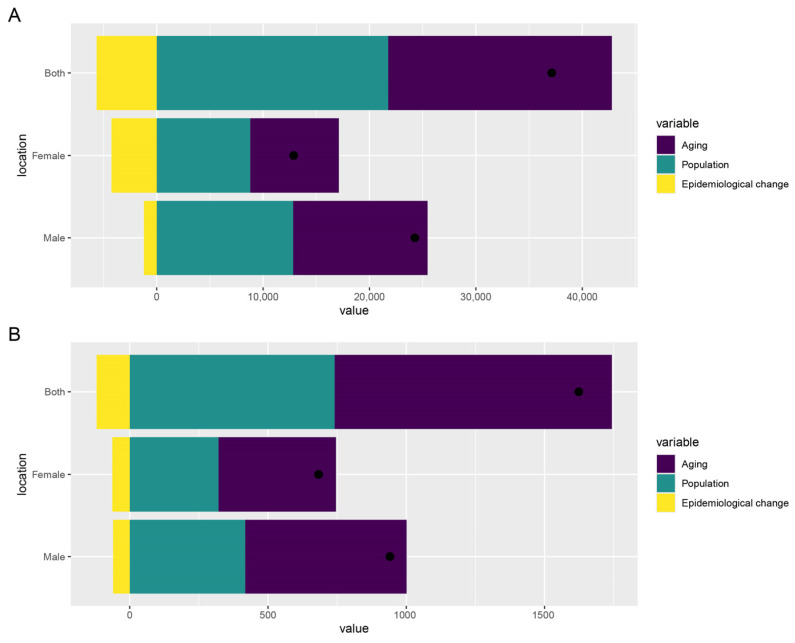
Decomposition of changes in the number of DALYs (**A**) and deaths (**B**) due to mesothelioma in China by sex from 1990 to 2023. The black dots represent the combined effects of these three factors.

Decomposition analysis revealed that, over the past 34 years, population growth was the most prominent contributor to the increase in mesothelioma DALYs in China, followed by population aging (Figure 6A). For DALYs, epidemiological change contributed −5646.96 (−15.21%), population aging contributed 21,021.92 (56.61%), and population growth contributed 21,757.03 (58.59%), resulting in an overall increase of 37,131.98 DALYs. The increase was 24,260.69 in males and 12,871.30 in females. For deaths, epidemiological change contributed −119.66 (−7.37%), population aging contributed 1002.59 (61.74%), and population growth contributed 740.93 (45.63%), resulting in an overall increase of 1623.86 deaths. The increase was 941.04 in males and 682.83 in females (Figure 6B).

### 3.7. Prediction Analysis of Mesothelioma Burden

**Figure 7:**
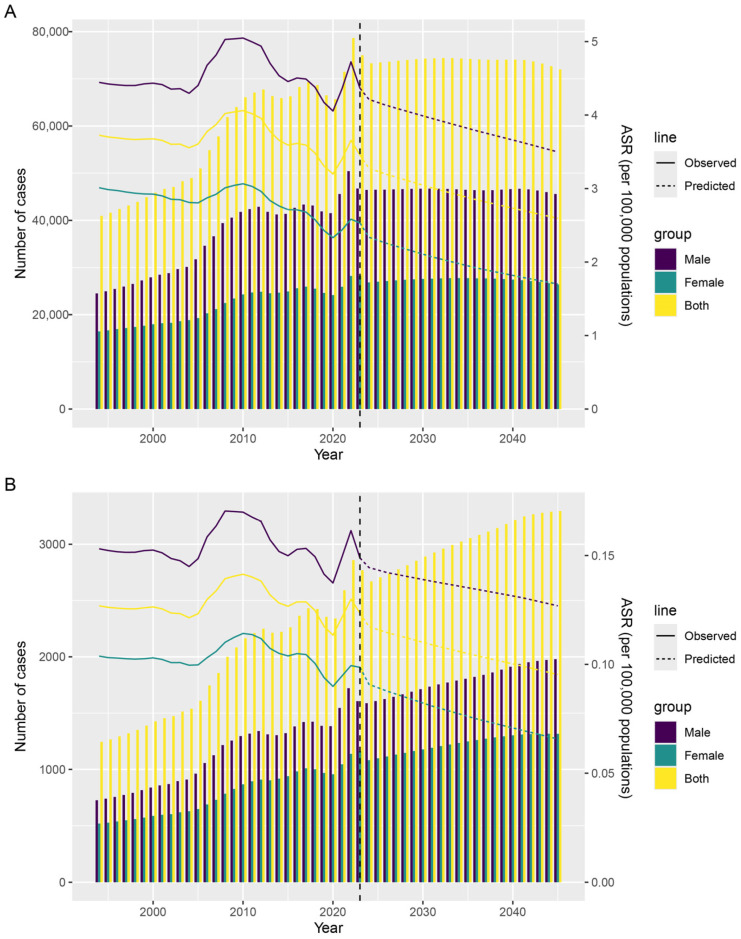
Projected trends in the burden of mesothelioma in China through 2045. (**A**) Projected number of DALYs and ASDR by year and sex. (**B**) Projected number of deaths and ASMR by year and sex.

Prediction analysis showed that, from 2024 to 2045, mesothelioma DALYs in China are expected to remain relatively stable, whereas deaths are projected to increase rapidly year by year (Figure 7A,B). In contrast, both ASDR and ASMR are expected to decline annually. By 2045, DALYs are projected to reach 45,589 in males, with an ASDR of 3.50 per 100,000 population, and 26,407 in females, with an ASDR of 1.70 per 100,000 population. Deaths are projected to reach 1979 in males, with an ASMR of 0.13 per 100,000 population, and 1316 in females, with an ASMR of 0.07 per 100,000 population.

## 4. Discussion

In this study, we assessed the burden of mesothelioma in China from 1990 to 2023 using GBD 2023 estimates. The results showed that the absolute burden of mesothelioma increased substantially over the past 34 years, including increases in prevalence, incidence, deaths, and DALYs. However, age-standardized fatal indicators did not increase in parallel, as ASMR remained broadly stable or showed a mild downward trend. This divergence suggests that the rising absolute burden should not be interpreted simply as a worsening of age-specific mortality risk. Instead, population aging, population growth, and the long latency of mesothelioma may have contributed substantially to the increasing number of affected individuals [21].

China was one of the world’s largest producers and consumers of asbestos from the 1950s through the early 2000s, with substantial use in construction, shipbuilding, manufacturing, and mining [22]. Although China banned crocidolite in 2002, other asbestos types remained in use, and asbestos-containing materials persist in older infrastructure [23]. The long latency (20–50 years) means that the burden observed during 1990–2023 likely reflects exposures from China’s peak industrial period.

The study period from 1990 to 2023 witnessed substantial improvements in China’s diagnostic capacity, including expanded availability of pathology services, improved imaging technologies (CT and MRI), and enhanced cancer registration through the National Cancer Registry [24]. These improvements may have contributed to increased detection and reporting of mesothelioma, particularly in later years. The rising ASIR and ASPR observed in our analysis may partially reflect improved diagnostic ascertainment and more complete cancer registration, rather than solely representing a true increase in disease occurrence. This diagnostic capacity improvement underscores the importance of continued investment in cancer surveillance infrastructure. Nevertheless, GBD data cannot directly distinguish between true changes in disease occurrence, diagnostic improvement, survival changes, or registry effects. Therefore, these explanations should be interpreted cautiously.

The burden of mesothelioma was consistently higher among males than females. This sex difference may be associated with greater historical occupational exposure among men in industries such as construction, mining, shipbuilding, manufacturing, insulation work, and industrial maintenance, where asbestos or asbestos-containing materials may have been used [2]. However, female mesothelioma should not be overlooked, as environmental exposure, household contact with contaminated work clothing, and other nonoccupational exposure pathways may also contribute to disease occurrence.

Age-specific analysis showed that mesothelioma burden was mainly concentrated among middle-aged and older adults, especially after 55 years of age. This finding is consistent with the long latency of mesothelioma and indicates that current disease burden may reflect exposures that occurred many years earlier. The concentration of cases and deaths among older adults also has important implications for healthcare planning, as older patients often have more comorbidities and may require multidisciplinary treatment, symptom control, supportive care, and palliative care [4,6,25].

Decomposition analysis showed that population aging and population growth were the main drivers of increasing deaths and DALYs, whereas epidemiological change contributed negatively. This finding further supports the interpretation that the rise in absolute fatal burden was largely driven by demographic transition. However, epidemiological change is a composite residual component and should not be interpreted as direct evidence of any single factor, such as improved treatment, reduced exposure, or better diagnosis. It may reflect combined changes in exposure patterns, disease detection, survival, registry quality, and GBD modeling assumptions [26].

The projection analysis suggested that mesothelioma deaths in China may continue to increase from 2024 to 2045, whereas ASDR and ASMR may decline. This pattern indicates that China may face increasing healthcare demand related to mesothelioma despite possible improvement in age-standardized fatal burden. However, these projections are based on historical trends and should be interpreted as scenario-based estimates rather than definitive forecasts [27]. Future changes in asbestos control, diagnostic capacity, cancer registration, treatment access, and survival may alter the projected burden.

The patterns observed in China share similarities with and differences from other countries [28,29]. In many high-income countries (US, Australia, Western Europe), mesothelioma incidence and mortality rates have plateaued or declined following comprehensive asbestos bans implemented from the 1970s–2000s [30,31]. Australia, which implemented a comprehensive ban in 2003, has seen declining incidence in younger cohorts [32]. China’s continued increases in absolute case numbers likely reflect later adoption of asbestos controls. Japan experienced a mesothelioma peak in the 2000s with gradual decline thereafter—a pattern that may foreshadow China’s future trajectory if current asbestos-control policies are maintained [33]. The male predominance and concentration among older adults observed in China are consistent with global patterns, reflecting historically male-dominated occupational exposures. However, the proportion of female cases in China may warrant particular attention, as non-occupational and environmental exposures may be relatively more important in settings with limited occupational health protections.

This study has several limitations. First, the analysis was based on GBD 2023 modelled estimates rather than individual-level registry or hospital data, and the results may be affected by data availability, coding accuracy, disease misclassification, and model assumptions. Second, GBD data do not provide detailed information on asbestos exposure, occupational history, tumor site, histological subtype, stage, treatment, or survival, limiting causal interpretation. Third, decomposition analysis cannot identify the specific mechanisms underlying epidemiological change. Fourth, future projections may change if exposure control, diagnostic capacity, cancer registration, or treatment strategies improve.

These findings and limitations highlight important gaps in the current national surveillance infrastructure. Addressing these gaps is essential to enable future analyses based on directly observed data rather than predominantly on model-based estimates. First, surveillance should be strengthened among older adults and individuals with potential occupational or environmental asbestos exposure. Second, detailed exposure histories should be systematically collected to improve risk identification. Third, pathological diagnosis should be standardized because mesothelioma is rare and can be difficult to distinguish from other pleural malignancies. Fourth, occupational and environmental prevention remains essential to reduce future disease occurrence. Finally, multidisciplinary care should be emphasized for older patients with mesothelioma [34].

## 5. Conclusions

In conclusion, the absolute burden of mesothelioma in China increased markedly from 1990 to 2023, while age-standardized fatal burden remained stable or declined slightly. Population aging and population growth were the main drivers of increasing deaths and DALYs. Broader policy implications, including strengthening asbestos exposure surveillance and enhancing multidisciplinary care, while consistent with the observed burden patterns, extend beyond the direct scope of this descriptive epidemiological analysis. These recommendations are informed by the established literature on mesothelioma prevention and management and should be interpreted as contextually relevant policy directions rather than evidence-based conclusions directly derived from our trend analysis.

## Figures and Tables

**Table 1:** All-age numbers and age-standardized rates of prevalence, incidence, mortality, and disability-adjusted life years (DALYs) for mesothelioma in China, 1990–2023.

Indicator	Sex	1990_Cases (95% UI)	2023_Cases (95% UI)	Percentage Change	1990_ASR_per 100,000 (95% UI)	2023_ASR_per 100,000 (95% UI)	EAPC (95% CI)
Prevalence	Both	1607.49 (1219.5–2116.23)	4640.31 (3545.91–6025.91)	1.89	0.17 (0.13–0.22)	0.21 (0.16–0.27)	0.83 (0.65–1.01)
	Female	571.29 (317.69–917.57)	1314.25 (704.11–2047.05)	1.3	0.12 (0.07–0.19)	0.11 (0.06–0.18)	−0.06 (−0.23–0.1)
	Male	1036.2 (709.86–1457.48)	3326.06 (2365.67–4644.55)	2.21	0.22 (0.15–0.31)	0.3 (0.22–0.42)	1.23 (1.03–1.42)
Incidence	Both	1237.85 (941.01–1628.9)	3097.28 (2370–3913.1)	1.5	0.13 (0.1–0.18)	0.14 (0.11–0.18)	0.2 (0.02–0.37)
	Female	500.92 (290.53–798.24)	1179.65 (625.74–1807.7)	1.35	0.11 (0.06–0.17)	0.1 (0.05–0.16)	−0.15 (−0.32–0.02)
	Male	736.93 (515.14–1041)	1917.63 (1403.23–2525.88)	1.6	0.16 (0.11–0.23)	0.18 (0.13–0.23)	0.36 (0.18–0.54)
DALYs	Both	37,848.71 (28,703.04–50,106.38)	74,980.69 (57,750.03–95,028.23)	0.98	3.81 (2.89–5.04)	3.46 (2.67–4.36)	−0.21 (−0.38–−0.03)
	Female	15,379.21 (8851.78–24,571.03)	28,250.51 (14,958.01–42,995.1)	0.84	3.11 (1.81–4.92)	2.53 (1.34–3.89)	−0.61 (−0.77–−0.45)
	Male	22,469.49 (15,621.34–31,968.27)	46,730.18 (34,599.89–62,472.31)	1.08	4.52 (3.15–6.44)	4.36 (3.22–5.92)	0.03 (−0.16–0.21)
Death	Both	1144.73 (864.61–1520.83)	2768.59 (2071.24–3498.68)	1.42	0.13 (0.1–0.17)	0.12 (0.09–0.16)	−0.03 (−0.2–0.14)
	Female	479.48 (277.82–766.51)	1162.3 (614.69–1801.24)	1.42	0.11 (0.06–0.17)	0.1 (0.05–0.15)	−0.14 (−0.31–0.03)
	Male	665.25 (463.37–952.77)	1606.29 (1172.09–2107.48)	1.41	0.16 (0.11–0.22)	0.15 (0.11–0.2)	−0.03 (−0.21–0.14)

## Data Availability

The datasets presented in this study can be found online (http://ghdx.healthdata.org/gbd-results-tool, accessed on 12 March 2026). Further information can be directed to the corresponding authors.
